# Association of healthy lifestyle with risk of obstructive sleep apnea: a cross-sectional study

**DOI:** 10.1186/s12890-021-01818-7

**Published:** 2022-01-11

**Authors:** Xueru Duan, Jun Huang, Murui Zheng, Wenjing Zhao, Lixian Lao, Haiyi Li, Zhiwei Wang, Jiahai Lu, Weiqing Chen, Hai Deng, Xudong Liu

**Affiliations:** 1 Department of Epidemiology, School of Public Health, Sun Yat-Sen University, 7/F, Public Health Building, No.74, Zhongshan Second Road, Guangzhou, 510080 China; 2 School of Public Health, Guangdong Pharmaceutical University, No. 283 Jianghai Avenue, Haizhu District, Guangzhou, 510310 China; 3 Department of Geriatrics, Institute of Geriatrics, Guangdong Provincial People’s Hospital, Guangzhou, China; 4 Guangzhou Center for Disease Control and Prevention, Guangzhou, China; 5 School of Public Health and Emergency Management, Southern University of Science and Technology, Shenzhen, China; 6 Department of Cardiology, Guangdong Cardiovascular Institute, Guangdong Provincial People’s Hospital/Guangdong Academy of Medical Science, 5/F, Ying Tung Building, No.106, Zhongshan Second Road, Guangzhou, 510080 China; 7 Shantou University Medical College, Shantou, China

**Keywords:** Lifestyle, Healthy lifestyle score, Obstructive sleep apnea

## Abstract

**Background:**

No studies investigated the whole effect of modifiable lifestyle factors on OSA risk. This study aimed to examine the individual and combined effects of lifestyle factors on OSA risk among Chinese adults.

**Methods:**

This cross-sectional study included 9733 participants aged 35 to 74 years from the baseline survey of Guangzhou Heart Study. OSA was evaluated by Berlin Questionnaire. The healthy lifestyle score (HLS), representing the overall effect of lifestyles, was derived from seven lifestyle factors: active smoking, passive smoking, alcohol, diet, waist-hip ratio, leisure-time physical activity, and mental status. Odds ratio (OR) with 95% confidence interval (CI) was calculated using the multivariate logistic regression model.

**Results:**

8107 participants were divided into the non-OSA group and 1626 participants into the OSA group. No passive smoking (OR 0.83, 95% CI 0.74–0.94), healthy waist-hip ratio (OR 0.67, 95% CI 0.58–0.77) and healthy mental status (OR 0.45, 95% CI 0. 29–0.73) were associated with a reduced risk of OSA after adjusting for confounders, while others not. Participants with higher HLS were negatively associated with OSA risk (*P*_-trend_ < 0.001). In comparison to the participants with 0–3 HLS, the OR for participants with 4, 5, 6, and 7 HLS was 0.68 (95% CI 0.56–0.84), 0.71 (95% CI 0.59–0.86), 0.62 (95% CI 0.51–0.76) and 0.49 (95% CI 0.37–0.65) after adjusting for confounders. Every 1-score increment of HLS was associated with a 13% lower risk of OSA.

**Conclusions:**

The results suggest that HLS reflecting the combined effect of multiple-dimensional lifestyle factors was inversely associated with OSA risk. Preventive strategies integrating multiple lifestyle factors may provide a more feasible approach for OSA prevention.

**Supplementary Information:**

The online version contains supplementary material available at 10.1186/s12890-021-01818-7.

## Background

Obstructive sleep apnea (OSA) is an increasingly common sleep disorder characterized by repeated collapse of the upper airway during sleep, resulting in recurrent oxyhemoglobin desaturation and periodic reductions or cessations in ventilation, with consequent hypoxia, hypercapnia, sleep fragmentation, or arousals from sleep [1, 2]. The prevalence of OSA was escalating worldwide recently and presented prodigious regional disparity with 9–38% in Europe and North America and 8.8–24.2% in China [2, 3]. It was estimated that a 5-year incidence of OSA was about 7–11% in middle-aged adults [2]. Evidence from cohort studies indicated that undiagnosed OSA, with or without symptoms, was an important contributor to cardiovascular, metabolic, and neurocognitive effects, as well as increased likelihood of motor vehicle accidents and diminished quality of life [4]. Nearly one billion adults aged 30–69 years worldwide were estimated to have OSA with 425 million requiring treatment, generating a high economic and social burden [5].

Several risk factors have been demonstrated to be associated with the occurrence and development of OSA, especially overweight, male, older age, anomalies of craniofacial structure, and hereditary factors [6, 7]. Recent studies have shown that lifestyle factors, including aerobic physical activity, diet, smoking, and alcohol drinking, were correlated with the occurrence and severity of OSA. Independent of known risk factors for OSA, increased levels of physical activity, including walking, were associated with a prevalence of OSA [8]. Simpson et al. found that low levels of occupational activity and leisure exercise were associated with moderate-severe OSA in a study with case–control design [9]. Several population-based studies have demonstrated alcoholics and smokers were more likely to have high-risk OSA [10–12]. A less healthy dietary profile with lower whole-grain intake and increased red/processed meat consumption was linked with moderate-to-more severe OSA, which were partially explained by reductions in slow-wave sleep, consequently leading to increased fragmented sleep and adiposity [13]. Besides, psychological status was also found to play an important role in the occurrence and development of OSA [14, 15]; individuals with psychiatric disorders, especially major depressive disorder, were associated with elevated levels of OSA [15].

Lifestyle behaviors often coexist and may play an interactive effect. Several epidemiological studies constructed lifestyle indices to capture the overall role of multi-dimensional lifestyle factors on health issues [16–18]. However, to date, most present studies on OSA focus on a single-dimension lifestyle factor (mainly exercise or diet), and no one considered the overall effect of multi-dimension lifestyles on OSA. Moreover, the benefits of lifestyle intervention in the management of OSA patients are highlighted, but the effect of primary prevention for OSA by healthy lifestyle is neglected.

Accordingly, based on the guideline for primary care of adult obstructive sleep apnea [19] and prior evidence [20, 21], we developed a healthy lifestyle index that contained seven potential modifiable lifestyle factors—active smoking, passive smoking, alcohol, diet, waist-hip ratio, leisure-time physical activity, and mental status, to examine the combined effect of these lifestyle factors on OSA among Chinese adults.

## Study design and methods

### Setting and subjects

**Fig. 1:**
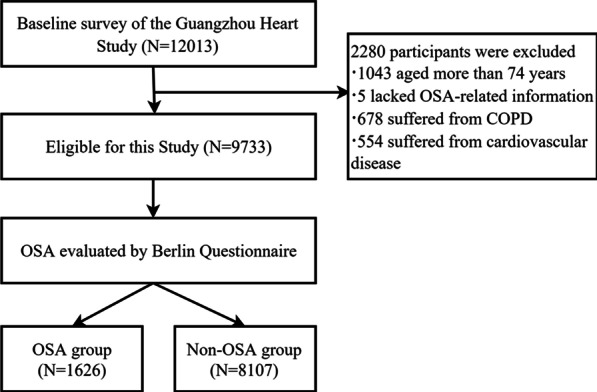
The flow chart for selecting participants

The data of this study was derived from the baseline survey of Guangzhou Heart Study (GZHS), an ongoing population-based prospective cohort study. The GZHS baseline survey was conducted from 2015 to 2017, and 12,013 subjects aged 35 years or above and permanently residing in Guangzhou were recruited by the multi-stage sampling method [22, 23]. Detailed information about GZHS has been reported in our previous reports [22, 23]. Among 12,013 participants, 2280 subjects were excluded due to the following conditions: more than 74 years old (n = 1043), lack of OSA-related data (n = 5), suffering from the chronic obstructive pulmonary disease (n = 678) or cardiovascular disease (n = 554). A detailed flow chart for selecting participants is shown in Fig. 1. Overall, 9733 participants were included for further analysis. This study was approved by the Ethical Committee of Guangdong Provincial People's Hospital and Ethical Review Committee for Biomedical Research, School of Public Health, Sun Yat-sen University. The study was performed in accordance with the Declaration of Helsinki and written informed consent was obtained from each participant before they joined in the study.

### OSA ascertainment

OSA was determined by the Berlin Questionnaire (BQ), a tool developed for assessing OSA risk in the primary care settings with the sensitivity of 77% and the specificity of 44% among the general population [21]. BQ contained 10 questions in three categories: snoring and cessation of breathing (category 1), symptoms of excessive daytime sleepiness (category 2), body mass index (BMI), and hypertension (category 3). High risk in category 1 and category 2 was defined by persistent symptoms (> 3–4 times/week) in at least two questions of each category and in category 3 was defined by the history of hypertension or BMI higher than 30 kg/m^2^. Subjects who were considered high-risk for OSA should be at high risk at least two categories and were distributed to the OSA group, otherwise, subjects were considered low-risk for OSA and distributed to the non-OSA group [24].

### Assessment of lifestyle factors

Questionnaires conducted with a face-to-face approach and physical examination were used to collect information of seven about lifestyle factors. A structured questionnaire was adopted to collect exposure information of smoking and alcohol drinking. Each subject was asked to report their current and prior smoking status. Participants were classified as nonsmokers if they had never smoked, as former smokers if they ever smoked but quitted smoking currently, as current smokers if they smoked frequently or occasionally when being recruited into the study. Besides, participants were required to answer whether they had exposed to smoke exhaled by other people in daily life or in workplace; if they answered “Yes”, then the frequency of exposure for more than 15 min per day (almost every day, ≥ three days per week, one to three days per week, less than one day per week, and unknown) was further required to answer. Active smoking was defined as current smoking at the time of recruitment to the study and passive smoking was defined as exposure to the smoke exhaled from smokers more than 15 min at least one day per week [25]. Information on alcohol consumption was also assessed and participants were required to report their current status of alcohol drinking: “frequent drinking”, “occasional drinking” and “never drinking or alcohol cessation”. Occasional or frequent alcohol consumption was considered as alcohol drinking.

Dietary information was obtained by a 22-item food frequency questionnaire (FFQ) and each participant was required to report the intake frequency of each food item (< once per month, 1–3 times per month, 1–3 times per week, 4–6 times per week and ≥ once per day) over the past year. A total of 12 major food items in FFQ were used to establish a diet quality score based on the Chinese Dietary Guidelines (2016) [26] and Guideline for primary care of adult obstructive sleep apnea (2018) [19]. Fruit and vegetables were separately assigned one point if they were consumed more than three times per week; whole grains, legumes, nuts, dairy, poultry, and fish were separately assigned one point if they were consumed at least once per week; red meat was assigned one point if it was consumed less than once per week; high-salt foods, fried foods, and sugary beverages were separately assigned one point if they were not consumed or consumed less than once per month. If the intake frequency of one food item did not meet the corresponding criteria aforementioned, a point of 0 was assigned to this item. Accordingly, the diet quality score ranged from 0 (lowest) to 12 (highest). A healthy diet was defined as the diet quality score higher than the median value, namely the score ≥ 7.

Waist-hip ratio (WHR) was included as an indicator of abdominal obesity, because the distribution of abdominal fat accumulation had a stronger relationship with obesity-related diseases when compared with the body fat distribution [27]. Waist and hip circumference were measured three times in succession using standard instruments. The waist-hip ratio was calculated as the mean of waist circumference divided by the mean of hip circumference. The waist-hip ratio of ≥ 0.90 for males and of ≥ 0.85 for females was defined as abdominal obesity and considered unhealthy [28].

Leisure-time physical activity (LTPA) was assessed by a modified Global Physical Activity Questionnaire [29]. The total volume of LTPA was defined as the sum of the volume of eight general categories of most common activities including stroll, Tai Chi/Qigong, brisk walking/health exercises/Yangko, long-distance running/aerobics dancing, swimming, ball games (basketball, table tennis, badminton, et al.), bicycling, and housework. The volume of each activity was calculated by multiplying the activity duration by frequency and then by the intensity (quantified by the value of metabolic equivalent, MET). Detailed evaluation of the volume of physical activity was shown in our previous report [22]. According to World Health Organization (WHO) guidelines on physical activity, adults should do at least 150–300 min of moderate-intensity aerobic physical activity, or at least 75–150 min of vigorous-intensity aerobic physical activity, or an equivalent combination of moderate- and vigorous-intensity activity throughout the week [30]; this means performing the activities with at least 10.0 MET-hours/week to achieve the minimum level of the recommended standard.

Mental status including depression and anxiety was measured by the Center for Epidemiologic Studies Depression Scale (CES-D) and Zung’s self-rating anxiety scale (SAS), respectively [31, 32]. The range of CES-D scores was 0 to 60, with a threshold of 16 or greater being considered as depression. The SAS index score ranged from 25 to 100 with a threshold of 45 or greater being considered as having anxiety. The participant who had either depression or anxiety was identified as having an unhealthy mental status.

### Derivation of the healthy lifestyle score

The healthy lifestyle score (HLS) was created based on seven modifiable lifestyle factors: active smoking, passive smoking, alcohol, diet, waist-hip ratio, leisure-time physical activity, and mental status. Each lifestyle factor was dichotomized as healthy or unhealthy and each factor was then assigned a score of 0 and 1 for unhealthy and healthy, respectively. Healthy criteria for lifestyle factors were: no active smoking, no passive smoking, no current alcohol drinking, having a healthy diet (the diet quality score ≥ 7), having a healthy weight (WHR < 0.90 in males or < 0.85 in females), being physically active (meeting the WHO guideline on physical activity), and having a healthy mental status (having neither depression nor anxiety). More details can be seen in Additional file 1: Table S1. HLS was the total sum of the score of seven lifestyle factors. Consequently, the healthy lifestyle score ranged from zero (least healthy) to seven (healthiest) points.

### Potential confounding factors

The structured questionnaire aforesaid and physical examination were also used to collect information of demographic characteristics and history of diseases, including age (years), sex (male, female), education (primary school or lower, junior high school, senior high school, and college or above), marital status (married and others), retirement status (yes, no), hypertension (physician-diagnosed, or systolic blood pressure ≥ 140 mmHg, or diastolic blood pressure ≥ 90 mmHg), diabetes (physician-diagnosed, or fasting blood glucose ≥ 7.0 mmol/L) [33] and dyslipidemia (physician-diagnosed, or serum cholesterol of ≥ 5.2 mmol/L, or low density lipoprotein cholesterol of ≥ 3.4 mmol/L, or triglyceride of ≥ 1.7 mmol/L) [34]. Height and weight were measured for three consecutive times, and BMI was calculated as mean of weight divided by mean of height squared (kg/m^2^). The mean of three measurements of neck circumference was calculated (cm).

### Statistical analysis

Kolmogorov–Smirnov test was used to examine the normality. The continuous variables with normal distribution were represented by mean and standard deviation (SD), and the continuous variables with non-normal distribution were represented by median and quartile range (IQR). The distribution of categorical variables was expressed as frequency and percentage. The distribution difference of demographic characteristics, history of diseases, and other covariates between the non-OSA group and the OSA group was evaluated by t-test, or Wilcoxon rank-sum test, or chi-square test, correspondingly. Cochran–Armitage trend test was used to examine whether the distribution of HLS between the non-OSA group and the OSA group was significant. The associations between HLS and its components were manifested by the spearman correlation coefficient (r_s_). Logistic regression was used to estimate unadjusted and adjusted odds ratios (ORs) and 95% confidence intervals (CIs) to demonstrate the individual and overall impact of lifestyle factors on OSA risk.

Stratified analyses were performed by sex, retirement status, and history of dyslipidemia. The multiplicative interaction of HLS with sex, retirement status, and history of dyslipidemia was examined respectively by using the likelihood ratio test, with a comparison of the likelihood scores of the two models with and without the interaction items.

Several sensitivity analyses were conducted to examine the robustness of our results. Each of the seven lifestyle factors was excluded in turn from HLS and repeated analyses were conducted to examine the stability of the HLS. The neck circumference was further adjusted in the multivariate model to consider the potential influence of fluid accumulation in the neck on OSA. Repeated analyses were also done by using STOP-Bang Questionnaire [35] to determine OSA, by using median in place of WHO-recommended cut-off value of LTPA in generating HLS on account of the fact that the volume of LTPA met the WHO recommendations for most participants (85.7%), by excluding subjects aged 65 years or above to rule out the effects of age-related factors, and by excluding subjects with BMI of less than 18.5 kg/m^2^ to rule out unknown bias brought by underweight.

All analyses were performed with R software (version 3.6.3). Two-sided *P* values were computed and *α* less than 0.05 was considered significant.

## Results

A total of 9733 participants were included in this study, of which 8107 participants (83.29%) were divided into the non-OSA group and 1626 participants (16.71%) into the OSA group. The means of age (58.53 vs. 55.48 years), BMI (26.34 vs. 23.54 kg/m^2^), waist-hip ratio (0.92 vs. 0.87) and neck circumference (36.59 vs. 34.17 cm) in the OSA group were all larger than those in the non-OSA group. In comparison to the non-OSA participants, participants with OSA were more likely to be male and married, to have a lower level of education, to have diabetes and dyslipidemia, to be retirees, to smoke (whatever actively or passively) or drink alcohol, to have worse mental status, were less likely to have an active leisure-time physical activity, to have a higher dietary quality score or healthy lifestyle score (Table 1). In Additional file 1: Table S2, seven individual lifestyle factors were all strongly correlated with HLS (all *P* < 0.001).

**Table1:** Baseline characteristics of the participants

Characteristic	Non-OSA group (N = 8107)	OSA group (N = 1626)	*P*
Age, years, mean (S.D.)	55.48 (9.98)	58.53 (8.93)	< 0.001*
Sex, N (%)			< 0.001^†^
Male	2469 (30.45)	804 (49.45)	
Female	5638 (69.55)	822 (50.55)	
Education, N (%)			0.003^‡^
Primary school or lower	2879 (35.51)	590 (36.28)	
Junior high school	2081 (25.67)	397 (24.42)	
Senior high school	2043 (25.20)	462 (28.41)	
College or above	1104 (13.62)	177 (10.89)	
Marital status, N (%)			< 0.001^†^
Married	7099 (87.57)	1476 (90.77)	
Others	1008 (12.43)	150 (9.23)	
Active smoking, N (%)			< 0.001^†^
No	6934 (85.53)	1292 (79.46)	
Yes	1173 (14.47)	334 (20.54)	
Passive smoking, N (%)			0.004 ^†^
No	5071 (62.55)	955 (58.73)	
Yes	3036 (37.45)	671 (41.27)	
Alcohol, N (%)			< 0.001^†^
Never or former drinking	6404 (78.99)	1168 (71.83)	
Occasional or frequent drinking	1703 (21.01)	458 (28.17)	
Dietary quality score, mean (S.D.)	7.43 (1.78)	7.19 (1.83)	< 0.001*
Waist-hip ratio, mean (S.D.)	0.87 (0.07)	0.92 (0.06)	< 0.001*
Leisure-time physical activity, MET-h/week, median (IQR)	35.70 (17.80, 59.20)	34.70 (15.00, 58.80)	< 0.001^§^
Mental status, N (%)			0.100 ^†^
Unhealthy	96 (1.18)	28 (1.72)	
Healthy	8011 (98.82)	1598 (98.28)	
Body mass index, kg/m^2^, mean (S.D.)	23.54 (3.26)	26.34 (3.84)	< 0.001*
Neck circumference, cm, mean (S.D.)	34.17 (3.05)	36.59 (3.45)	< 0.001*
Hypertension, N (%)			< 0.001^†^
No	5669 (69.93)	157 (9.66)	
Yes	2438 (30.07)	1469 (90.34)	
Diabetes, N (%)			< 0.001^†^
No	7509 (92.62)	1446 (88.93)	
Yes	598 (7.38)	180 (11.07)	
Retirement status, N (%)			< 0.001^†^
No	4149 (51.18)	683 (42.00)	
Yes	3958 (48.82)	943 (58.00)	
Dyslipidemia, N (%)			< 0.001^†^
No	2497 (30.80)	414 (25.46)	
Yes	5610 (69.20)	1212 (74.54)	
Healthy lifestyle score, N (%)			< 0.001^‡^
0–3	684 (8.43)	272 (16.73)	
4	1166 (14.38)	303 (18.63)	
5	2310 (28.49)	514 (31.61)	
6	2772 (34.19)	438 (26.94)	
7	1175 (14.49)	99 (6.09)	

*P value for t-test

^†^*P* for chi-square test

^‡^*P* for Cochran–Armitage trend test

^§^*P* for Wilcoxon rank-sum test

For individual lifestyle factors, no passive smoking (OR 0.83, 95% CI 0.74–0.94), healthy waist-hip ratio (OR 0.67, 95% CI 0.58–0.77) and healthy mental status (OR 0.45, 95% CI 0. 29–0.73) were associated with a lower risk of OSA, while others not (Table 2). When considering the overall effect of all healthy lifestyle factors after adjusting for potential confounders, a lower risk of OSA was significantly associated with an increment of HLS (*P*_-trend_ < 0.001) (Table 3). In comparison to the participants with 0–3 HLS, the OR for participants with 4, 5, 6, and 7 HLS was 0.68 (95% CI 0.56–0.84), 0.71 (95% CI 0.59–0.86), 0.62 (95% CI 0.51–0.76), and 0.49 (95% CI 0.37–0.65), respectively, after adjusting for potential confounders. Every 1-point increment of HLS was associated with 13% (OR 0.87, 95% CI 0.83–0.92) reduced risk of OSA.

**Table 2:** Association between each lifestyle factor and OSA risk

Lifestyle factor score	N*		Effect		
	Non-OSA group	OSA group	Unadjusted OR (95% CI)	Adjusted OR (95% CI)^†^	Adjusted OR (95% CI)^‡^
Active smoking					
Yes	1173	334	1.00	1.00	1.00
No	6934	1292	0.65 (0.57, 0.75)	0.68 (0.57, 0.80)	0.85 (0.71, 1.01)
Passive smoking					
Yes	3036	671	1.00	1.00	1.00
No	5071	955	0.85 (0.76, 0.95)	0.85 (0.76, 0.96)	0.83 (0.74, 0.94)
Alcohol					
Yes	1703	458	1.00	1.00	1.00
No	6404	1168	0.68 (0.60, 0.77)	0.97 (0.85, 1.11)	0.93 (0.81, 1.07)
Diet					
Unhealthy	2447	570	1.00	1.00	1.00
Healthy	5660	1056	0.80 (0.72, 0.90)	0.92 (0.82, 1.04)	0.95 (0.84, 1.08)
Waist-hip ratio					
Unhealthy	4282	1246	1.00	1.00	1.00
Healthy	3825	380	0.34 (0.30, 0.39)	0.36 (0.31, 0.41)	0.67 (0.58, 0.77)
Leisure-time physical activity					
Unhealthy	1123	273	1.00	1.00	1.00
Healthy	6984	1353	0.80 (0.69, 0.92)	0.93 (0.80, 1.09)	0.94 (0.80, 1.11)
Mental status					
Unhealthy	96	28	1.00	1.00	1.00
Healthy	8011	1598	0.68 (0.45, 1.06)	0.61 (0.40, 0.96)	0.45 (0.29, 0.73)

*N represents sample size for non-OSA group or for OSA group

^†^Adjustment for age, sex, education, marital status, diabetes, dyslipidemia, retirement status, and all other healthy lifestyle factors

^‡^Additional adjustment for body mass index

**Table 3:** Association between the healthy lifestyle score and OSA risk

Hlealthy lifestyle score	N*		Effect		
	Non-OSA group	OSA group	Unadjusted OR (95% CI)	Adjusted OR (95% CI)^†^	Adjusted OR (95% CI)^‡^
0–3	684	272	1.00	1.00	1.00
4	1166	303	0.65 (0.54, 0.79)	0.71 (0.59, 0.87)	0.68 (0.56, 0.84)
5	2310	514	0.56 (0.47, 0.66)	0.68 (0.56, 0.82)	0.71 (0.59, 0.86)
6	2772	438	0.40 (0.33, 0.47)	0.51 (0.42, 0.62)	0.62 (0.51, 0.76)
7	1175	99	0.21 (0.16, 0.27)	0.29 (0.22, 0.37)	0.49 (0.37, 0.65)
*P* for trend			< 0.001	< 0.001	< 0.001
Every 1-point increment			0.74 (0.71, 0.77)	0.79 (0.76, 0.83)	0.87 (0.83, 0.92)

*N represents sample size for non-OSA group or for OSA group

^†^Adjustment for age, sex, education, marital status, retirement status, diabetes and dyslipidemia

^‡^Additional adjustment for body mass index

In sensitivity analysis, seven repeated analyses were conducted by excluding one factor from HLS in turn and consistent protective effect by HLS was observed (Additional file 1: Table S3). Besides, repeated analyses also yielded similar results by using STOP-Bang Questionnaire to determine OSA, by using median in place of WHO-recommended cut-off value of LTPA in generating HLS, by excluding subjects aged 65 years or more, and by excluding participants with BMI less than 18.5 kg/m^2^, and by further adjustment for neck circumference (Additional file 1: Tables S4–S8).

In stratified analyses, a remarkably reduced risk of OSA was observed with the increase of HLS in both genders (Table 4), in both retirees and non-retirees (Table 5), and in subjects with and without dyslipidemia (Table 6). When comparing the highest with the lowest categories of HLS (7 vs. ≤ 3), the OR for male, female, retirees, non-retirees, subjects with dyslipidemia and subjects without dyslipidemia was 0.55 (95% CI 0.37–0.82), 0.38 (95% CI 0.24–0.62), 0.39 (95% CI 0.24–0.60), 0.66 (95% CI 0.45–0.97), 0.40 (95% CI 0.23–0.69), 0.55 (95% CI 0.40–0.75), respectively, after adjustment for confounders. However, by using the multiplicative interaction model, a significant interaction of HLS with retirement status (*P*_-interaction_ < 0.001) and history of dyslipidemia (*P*_-interaction_ < 0.001) but rather than with sex (*P*_-interaction_ = 0.463) was observed.

**Table 4:** Stratified analysis on the association between healthy lifestyle score and OSA risk by sex

Healthy lifestyle score	Male				Female			
	Non-OSA group	OSA group	Unadjusted OR (95% CI)	Adjusted OR (95% CI)*	Non-OSA group	OSA group	Unadjusted OR (95% CI)	Adjusted OR (95% CI)*
0–3	558	234	1.00	1.00	126	38	1.00	1.00
4	562	176	0.75 (0.59, 0.94)	0.71 (0.56, 0.91)	604	127	0.70 (0.47, 1.06)	0.55 (0.36, 0.87)
5	627	213	0.81 (0.65, 1.01)	0.78 (0.61, 0.99)	1683	301	0.59 (0.41, 0.88)	0.56 (0.37, 0.85)
6	500	144	0.69 (0.54, 0.87)	0.71 (0.55, 0.93)	2272	294	0.43 (0.30, 0.64)	0.48 (0.32, 0.73)
7	222	37	0.40 (0.27, 0.57)	0.55 (0.37, 0.82)	953	62	0.22 (0.14, 0.34)	0.38 (0.24, 0.62)
*P* for trend			< 0.001	0.150			< 0.001	0.010
Every 1-point increment			0.87 (0.82, 0.92)	0.89 (0.84, 0.95)			0.72 (0.67, 0.77)	0.86 (0.79, 0.93)

*Adjusted for age, education, marital status, diabetes, dyslipidemia, retirement status, and body mass index

**Table 5:** Stratified analysis on the association between healthy lifestyle score and OSA risk by retirement status

Healthy lifestyle score	Non-retirement				Retirement			
	Non-OSA group	OSA group	Unadjusted OR (95% CI)	Adjusted OR (95% CI)*	Non-OSA group	OSA group	Unadjusted OR (95% CI)	Adjusted OR (95% CI)*
0–3	482	188	1.00	1.00	202	84	1.00	1.00
4	638	149	0.60 (0.47, 0.76)	0.65 (0.50, 0.86)	528	154	0.70 (0.51, 0.96)	0.82 (0.59, 1.15)
5	1122	190	0.43 (0.35, 0.55)	0.64 (0.49, 0.83)	1188	324	0.66 (0.50, 0.87)	0.90 (0.66, 1.24)
6	1300	127	0.25 (0.20, 0.32)	0.52 (0.38, 0.69)	1472	311	0.51 (0.38, 0.68)	0.82 (0.60, 1.13)
7	607	29	0.12 (0.08, 0.18)	0.39 (0.24, 0.60)	568	70	0.30 (0.21, 0.42)	0.66 (0.45, 0.97)
*P* for trend			< 0.001	< 0.001			< 0.001	< 0.001
Every 1-point increment			0.66 (0.62, 0.70)	0.83 (0.77, 0.89)			0.79 (0.74, 0.84)	0.93 (0.87, 1.01)

*Adjusted for age, sex, education, marital status, diabetes, dyslipidemia, and body mass index

**Table 6:** Stratified analysis on the association between healthy lifestyle score and OSA risk by history of dyslipidemia

Healthy lifestyle score	Non-dyslipidemia				Dyslipidemia			
	Non-OSA group	OSA group	Unadjusted OR (95% CI)	Adjusted OR (95% CI)*	Non-OSA group	OSA group	Unadjusted OR (95% CI)	Adjusted OR (95% CI)*
0–3	192	58	1.00	1.00	492	214	1.00	1.00
4	288	90	1.03 (0.71, 1.51)	1.08 (0.71, 1.66)	878	213	0.56 (0.45, 0.69)	0.59 (0.47, 0.75)
5	680	133	0.65 (0.46, 0.92)	0.74 (0.50, 1.11)	1630	381	0.54 (0.44, 0.65)	0.71 (0.57, 0.89)
6	917	109	0.39 (0.28, 0.56)	0.55 (0.37, 0.84)	1855	329	0.41 (0.33, 0.50)	0.67 (0.53, 0.84)
7	420	24	0.19 (0.11, 0.31)	0.40 (0.23, 0.69)	755	75	0.23 (0.17, 0.30)	0.55 (0.40, 0.75)
*P* for trend			< 0.001	< 0.001			< 0.001	< 0.001
Every 1-point increment			0.69 (0.64, 0.75)	0.79 (0.71, 0.87)			0.76 (0.73, 0.80)	0.91 (0.86, 0.97)

*Adjusted for age, sex, education, marital status, diabetes, retirement status, and body mass index

## Discussion

To the best of our knowledge, this is the first large-scale population-based study in China to investigate both individual and overall effect of lifestyle factors on OSA risk. We found that HLS constituted by seven modifiable lifestyle factors (active smoking, passive smoking, alcohol, diet, waist-hip ratio, leisure-time physical activity, and mental status) was adversely associated with OSA risk. The stratified and sensitivity analyses yielded consistent results. These provided important evidence for adopting a healthy lifestyle to prevent OSA among the general population.

Plenty of studies have examined the association between individual component of HLS and OSA risk [14, 36–40], whereas up-to-date no study examined the overall effect of multiple lifestyle factors on the risk of OSA. Our study created a new variable of HLS, which reflected the overall effect of the seven most common modifiable lifestyle factors including active smoking, passive smoking, alcohol, diet, waist-hip ratio, leisure-time physical activity, and mental status. Our study found that HLS was negatively associated with the risk of OSA and three stratified analyses and five sensitivity analyses yielded similar consistent results, indicating that our results were robust and stable. Our findings on OSA were consistent with those reports on other sleep-related problems such as sleep disturbances [41] and sleep disruption [42], though the lifestyle components and overall effect indexes were different across these studies. Liu and colleagues defined five health-risk behaviors including current smoking, current drinking, unhealthy eating habits, insufficient leisure activities, and physical inactivity among the Chinese elderly, and found that the risk of sleep disturbances was positively correlated with the number of multiple health-risk behaviors [41]. A cross-sectional study among postmenopausal Iranian women found that the total score of ten lifestyle factors, including physical health, physical activity, weight control and nutrition, psychological health, spiritual health, social health, medications, and narcotics avoidance, illness prevention, accident prevention, and environmental health, was inversely related to sleep disruption [42].

The four most common used lifestyle factors as well as three new factors (passive smoking, waist-hip ratio, and mental status) were selected in our study to generate HLS, which can better reflect the overall lifestyle profile. In consistent with other studies on lifestyle integrating effect [16–18], four most common lifestyle components including active smoking, alcohol drinking, diet, and LTPA were also considered in our study. These four lifestyle factors were found to be highly correlated with the development of OSA [4, 13, 20]. Physical activity and diet, protected against OSA mainly by weight loss [13, 20, 37]. Overweight and obesity are widely recognized to be correlated with OSA and new prospective data showed that moderate weight gain in the general population might lead to an increase in the incidence and progression of OSA [43]. A meta-analysis showed that the single most effective way to reduce the incidence of OSA in a population is to prevent or reduce overweight and obesity [7]. Many studies have demonstrated physical activity- and diet-oriented lifestyle intervention plan played a leading role in weight loss [20, 44]. Reid’s study found that moderate-to-severe OSA was associated with a less healthy dietary profile on account of reduced slow-wave sleep [13]. Diet and exercise were likely complementary, with the former inducing weight loss and improving OSA severity and the latter improving general well-being [20]. Effective diet and exercise modification programs could yield weight loss in the long-term, and weight control was potentially the best nonmedical means of treating or arresting the OSA progression in clinical settings, and reducing the prevalence of OSA and its associated sequelae in a public health context [4]. Smoking and alcohol drinking are often considered as risk factors for OSA with limited studies conducted to analyze this association. Smoking could increase sleep instability and airway inflammation, which is linked to OSA [4, 45]. In addition, smoking-related airway inflammation and disease may increase vulnerability to OSA [4]. Alcohol ingestion has been demonstrated to acutely increase nasal and pharyngeal resistance in awake subjects with increased number and duration of hypopnea and apnea events; administration of alcohol near bedtime could imply an adverse acute impact on breathing during sleep [4, 11]. Hence, the control of smoking and alcohol drinking is of great significance for the prevention of OSA.

Among three new factors in generating HLS, a high household second-hand smoke exposure rate of 48.3% was found in China [25], and in our study exposure rate of passive smoking was 37.45% and 41.27% for the non-OSA group and the OSA group. Passive smokers might expose to hundreds of toxic and carcinogenic substances such as formaldehyde, benzene, and ammonia, which were related to the occurrence of serious respiratory diseases [25]. Therefore, passive smoking should be considered when assessing the smoking status of participants, apart from active smoking. The waist-hip ratio, an indicator of abdominal obesity, was even more strongly associated with obesity-related diseases. Increased abdominal fat appeared to be an independent predictor of risk in people who were not too high in BMI [28]. A review compared the predictive ability of BMI and waist-hip ratio for OSA and found that waist-hip ratio performed better than BMI because the accumulation of abdominal fat affected much more than peripheral fat as to the size and function of the upper airway [40]. Mental health is also an important component of lifestyle factors, and it is necessary to take the mental state into account when evaluating the overall effects of a healthy lifestyle. Current evidence suggested that depression can lead to difficulty falling asleep, frequent waking during the night and early morning, and a shortened rapid-eye-movement latency period [46]; persistent anxiety was independently associated with higher levels of sleep-disordered breathing [47]. In addition, depression and anxiety may bidirectionally be associated with OSA and they were often coexisted [14].

Among individual lifestyle factors, only passive smoking, waist-hip ratio, and mental status were associated with OSA risk, indicating the leading role of absence from second-hand smoke, weight loss, and healthy mental status in the protection of OSA as we have discussed above. Of note, the association between HLS and OSA risk was notably significant, even if not each lifestyle factor was associated with OSA risk. Stratified analysis by sex, retirement status, and history of dyslipidemia yielded consistent results with the main analysis that the risk of OSA de-escalated with the rise of HLS. Considering the higher prevalence of OSA in men, we conducted stratified analysis by sex and found that a healthy lifestyle played a protective effect against the occurrence of OSA among both males and females, especially for females. The reason for this disparity may be due to the hormonal influence on breathing control and upper airway muscle activation during sleep, resulting in 2 to 3 times more men are affected by OSA than premenopausal age-matched women [7]. The stratified analysis by retirement status identified that OSA risk decreased with increasing HLS among non-retired participants and HLS of more than 6 points was found to have a protective effect on OSA in the retired participants. This result was reasonable since the retirees tended to be older, and the physical damage from years of work negatively affected both women’s and men’s physical functioning in old age [48]. Retirement may indeed be associated with changes in lifestyle, but the impact may be heterogeneous depending on the type of lifestyle, lifestyle indicators, and the personal situation of the retiree [49]. LTPA seems to increase slightly after retirement in most studies, especially moderate-intensive physical activity, while for dietary habits, smoking, and alcohol drinking, there is not enough evidence to draw any conclusion [49]. Due to the consideration that dyslipidemia may lead to increased sympathetic activity, oxidative stress, insulin resistance, and endothelial dysfunction, thereby affecting the occurrence and development of OSA [50], the stratified analysis by the history of dyslipidemia was conducted and a similar protective effect was observed in both non-dyslipidemia and dyslipidemia groups.

There are several strengths. First, this is the first large-scale study among Chinese adults to investigate the association of combined effects of lifestyle factors with OSA risk and develop an indicator of HLS comprehensively considering physical and mental status. Our results provided useful clues for further longitudinal studies. Second, the multi-stage sampling method potentiated the representativeness of participants and the large sample size increased the statistic power. Third, the fact that multiple stratified analyses and sensitivity analyses displayed accordant results, indicating good internal consistency for the HLS evaluated.

Some limitations exist in this study as well. First, the cross-sectional design limited the ability of causal inference and was susceptible to bias. Second, the OSA assessment was based on the Berlin Questionnaire but not polysomnography, so we could not determine the severity of OSA. However, Berlin Questionnaire has been validated and specially developed for the primary care setting [21], which was suitable for this population-based study. We also used STOP-Bang Questionnaire to determine OSA in sensitivity analysis and then got consistent results, reflecting that our results are credible to a large degree. Third, the components of HLS and the cut-offs for each lifestyle factor were selected based on OSA-related clinical guidelines and without uniform criteria. Nevertheless, seven components were significantly correlated with HLS and no collinearity was found among these lifestyle factors. Moreover, we did seven repeated analyses by excluding each of the seven components of HLS each time and got similar results with the main analysis, demonstrating the stability of HLS. Fourth, a simplified diet quality score was used due to the lack of standard or recognized dietary scoring systems for OSA, which might not adequately clarify the complexity and reliability of diet. There is an urgent need to develop a dietary guideline specifically for OSA.

## Conclusions

The results suggest that HLS reflecting the combined effect of multiple-dimensional lifestyle factors was inversely associated with OSA risk. Preventive strategies integrating multiple lifestyle factors may provide a more feasible approach for OSA prevention.

## Supplementary Information

**Additional file 1: Table S1.** Description and scoring criteria of lifestyle factors. **Table S2.** The correlation between each lifestyle factor and healthy lifestyle score. **Table S3.** Association of every 1-unit increment of healthy lifestyle score with OSA risk after excluding each factor in turn. **Table S4**. Sensitivity analysis on the association between healthy lifestyle score and OSA risk after further adjusting for neck circumference. **Table S5**. Association between the healthy lifestyle score and OSA risk based on STOP-Bang Questionnaire. **Table S6.** Association between healthy lifestyle score and OSA risk by using median in place of WHO recommended cut-off value of LTPA in generating HLS. **Table S7.** Sensitivity analysis on the association between healthy lifestyle score and OSA risk after excluding participants aged 65 years and above. **Table S8**. Sensitivity analysis on the association between healthy lifestyle score and OSA risk after excluding participants with BMI less than 18.5 kg/m^2^

## Data Availability

The data used to support the findings of this study are available from the corresponding author upon request. A proposal with detailed description of study objectives and statistical analysis plan will be needed for evaluation of the reasonability of requests if someone requests data sharing.

## Abbreviations

BMI: Body mass index
BQ: Berlin questionnaire
CES-D: The Center for Epidemiologic Studies Depression Scale
CIs: Confidence intervals
FFQ: Food frequency questionnaire
GZHS: Guangzhou Heart Study
HLS: Healthy lifestyle score
LTPA: Leisure-time physical activity
MET: Metabolic equivalent
ORs: Odds ratios
OSA: Obstructive sleep apnea
SAS: Self-rating Anxiety Scale
WHO: World health organization
WHR: Waist-hip ratio
