# Supplementary material for: Association of healthy lifestyle with risk of obstructive sleep apnea: a cross-sectional study
Source: BMC Pulm Med. 2022 Jan 11;22:33. doi: 10.1186/s12890-021-01818-7 (PMC8751297; doi:10.1186/s12890-021-01818-7)
Supplement: Supplementary file 1 — Additional file 1: Table S1. Description and scoring criteria of lifestyle factors. Table S2. The correlation between each lifestyle factor and healthy lifestyle score. Table S3. Association of every 1-unit increment of healthy lifestyle score with OSA risk after excluding each factor in turn. Table S4. Sensitivity analysis on the association between healthy lifestyle score and OSA risk after further adjusting for neck circumference. Table S5. Association between the healthy lifestyle score and OSA risk based on STOP-Bang Questionnaire. Table S6. Association between healthy lifestyle score and OSA risk by using median in place of WHO recommended cut-off value of LTPA in generating HLS. Table S7. Sensitivity analysis on the association between healthy lifestyle score and OSA risk after excluding participants aged 65 years and above. Table S8. Sensitivity analysis on the association between healthy lifestyle score and OSA risk after excluding participants with BMI less than 18.5 kg/m2 [file 12890_2021_1818_MOESM1_ESM.docx]

**Supplemental material**

e-Table 1 Description and scoring criteria of lifestyle factors

e-Table 2 The correlation between each lifestyle factor and healthy lifestyle score

e-Table 3 Association of every 1-unit increment of healthy lifestyle score with OSA risk after excluding each factor in turn

e-Table 4 Sensitivity analysis on the association between healthy lifestyle score and OSA risk after further adjusting for neck circumference

e-Table 5 Association between the healthy lifestyle score and OSA risk based on STOP-Bang Questionnaire

e-Table 6 Association between healthy lifestyle score and OSA risk by using median in place of WHO recommended cut-off value of LTPA in generating HLS

e-Table 7 Sensitivity analysis on the association between healthy lifestyle score and OSA risk after excluding participants aged 65 years and above

e-Table 8 Sensitivity analysis on the association between healthy lifestyle score and OSA risk after excluding participants with BMI less than 18.5 kg/m^2^

e-Table 1 Description and scoring criteria of lifestyle factors

| Lifestyle factor | Classification | Point | Description |
| --- | --- | --- | --- |
| Active smoking | Yes | 0 | Active smoking: current smoking |
|  | No | 1 | No active smoking: never smoking or former smoking |
| Passive smoking | Yes | 0 | Passive smoking: exposure to the smoke exhaled from smokers more than 15min on at least 1 day per week |
|  | No | 1 | No passive smoking: not exposure to the smoke or the frequency of exposure to the smoke exhaled from smokers more than 15min less than 1 day per week |
| Alcohol | Yes | 0 | Occasional or frequent drinking |
|  | No | 1 | Never or former drinking |
| Diet | Unhealthy | 0 | Diet quality score < 7 |
|  | Healthy | 1 | Diet quality score ≥ 7 |
| Waist-hip ratio | Unhealthy | 0 | ≥ 0.90 in male; ≥ 0.85 in female |
|  | Healthy | 1 | < 0.90 in male; < 0.85 in female |
| Leisure-time physical activity | Unhealthy | 0 | Did not meet WHO guidelines on physical activity: less than 10.0 MET-h/week |
|  | Healthy | 1 | Meet WHO guidelines on physical activity: 10.0 MET-h/week or above |
| Mental status | Unhealthy | 0 | Suffer from depression or anxiety: CES-D score ≥ 16 or SAS index score ≥ 45 |
|  | Healthy | 1 | Did not suffer from depression and anxiety: CES-D score < 16 and SAS index score < 45 |

e-Table 2 The correlation coefficients between each lifestyle factor and healthy lifestyle score

| Lifestyle factor | r_s_^*^ | *P* |
| --- | --- | --- |
| Active smoking | 0.492 | <0.001 |
| Passive smoking | 0.498 | <0.001 |
| Alcohol | 0.476 | <0.001 |
| Diet | 0.512 | <0.001 |
| Waist-hip ratio | 0.386 | <0.001 |
| Leisure-time physical activity | 0.416 | <0.001 |
| Mental status | 0.097 | <0.001 |

^*^ r_s_ represents the Spearman correlation coefficient between individual lifestyle factor and healthy lifestyle score.

e-Table 3 Association of every 1-point increment of healthy lifestyle score with OSA risk after excluding each factor in turn

| Healthy lifestyle score |  | Effect | | |
| --- | --- | --- | --- | --- |
|  |  | Unadjusted OR (95% CI) | Adjusted OR (95% CI) ^†^ | Adjusted OR (95% CI) ^‡^ |
| Exclude active smoking |  | 0.70 (0.67, 0.74) | 0.72 (0.68, 0.76) | 0.83 (0.79, 0.88)) |
| Exclude passive smoking |  | 0.70 (0.67, 0.73) | 0.77 (0.73, 0.82) | 0.89 (0.84, 0.94) |
| Exclude alcohol |  | 0.71 (0.68, 0.75) | 0.76 (0.72, 0.80) | 0.86 (0.82, 0.91) |
| Exclude diet |  | 0.70 (0.67, 0.73) | 0.76 (0.72, 0.80) | 0.86 (0.81, 0.91) |
| Exclude waist-hip ratio |  | 0.83 (0.80, 0.87) | 0.91 (0.87, 0.96) | 0.91 (0.86, 0.96) |
| Exclude leisure-time physical activity |  | 0.71 (0.68, 0.74) | 0.77 (0.74, 0.81) | 0.87 (0.82, 0.91) |
| Exclude mental status |  | 0.74 (0.71, 0.77) | 0.79 (0.76, 0.83) | 0.88 (0.84, 0.93) |

^†^ Adjustment for age, sex, education, marital status, diabetes, dyslipidemia, retirement status, and all other healthy lifestyle factors.

^‡^ Additional adjustment for body mass index.

e-Table 4 Sensitivity analysis on the association between healthy lifestyle score and OSA risk after further adjusting for neck circumference

| Healthy lifestyle score | N^*^ | |  | Effect | | |
| --- | --- | --- | --- | --- | --- | --- |
|  | Non-OSA group | OSA group |  | Unadjusted OR (95% CI) | Adjusted OR (95% CI) ^†^ | Adjusted OR (95% CI) ^‡^ |
| 0-3 | 684 | 272 |  | 1.00 | 1.00 | 1.00 |
| 4 | 1166 | 303 |  | 0.65 (0.54, 0.79) | 0.71 (0.59, 0.87) | 0.69 (0.56, 0.85) |
| 5 | 2310 | 514 |  | 0.56 (0.47, 0.66) | 0.68 (0.56, 0.82) | 0.72 (0.59, 0.87) |
| 6 | 2772 | 438 |  | 0.40 (0.33, 0.47) | 0.51 (0.42, 0.62) | 0.63 (0.52, 0.78) |
| 7 | 1175 | 99 |  | 0.21 (0.16, 0.27) | 0.29 (0.22, 0.37) | 0.51 (0.38, 0.66) |
| *P* for trend |  |  |  | <0.001 | <0.001 | <0.001 |
| Every 1-point increment |  |  |  | 0.74 (0.71, 0.77) | 0.85 (0.81, 0.89) | 0.88 (0.84, 0.92) |

^*^ N represents sample size for non-OSA group or for OSA group.

^†^ Adjustment for age, sex, education, marital status, diabetes and dyslipidemia.

^‡^ Additional adjustment for neck circumference and body mass index.

e-Table 5 Association between the healthy lifestyle score and OSA risk based on STOP-Bang Questionnaire^*^

| Healthy lifestyle score | N^†^ | |  | Effect | | |
| --- | --- | --- | --- | --- | --- | --- |
|  | Non-OSA group | OSA group |  | Unadjusted OR (95% CI) | Adjusted OR (95% CI) ^‡^ | Adjusted OR (95% CI) ^ξ^ |
| 0-3 | 498 | 458 |  | 1.00 | 1.00 | 1.00 |
| 4 | 1046 | 423 |  | 0.44 (0.37, 0.52) | 0.69 (0.56, 0.84) | 0.66 (0.53, 0.82) |
| 5 | 2236 | 588 |  | 0.29 (0.24, 0.33) | 0.72 (0.59, 0.87) | 0.75 (0.61, 0.92) |
| 6 | 2778 | 432 |  | 0.17 (0.14, 0.20) | 0.5 (0.41, 0.62) | 0.59 (0.47, 0.74) |
| 7 | 1130 | 144 |  | 0.14 (0.11, 0.17) | 0.38 (0.29, 0.5) | 0.65 (0.48, 0.87) |
| *P* for trend |  |  |  | <0.001 | <0.001 | <0.001 |
| Every 1-point increment |  |  |  | 0.62 (0.60, 0.64) | 0.82 (0.78, 0.86) | 0.90 (0.85, 0.95) |

^*^The STOP-Bang Questionnaire includes eight dichotomous (yes/no) questions related to the clinical features of sleep apnea: snoring, tiredness, observed apnea, high blood pressure, BMI > 35 kg/m^2^, age > 50 years, neck circumference > 40 cm and male gender. For each question, answering “yes” scores 1, a “no” response scores 0, and the total score ranges from 0 to 8. Participants with a score of ≥ 3 were classified as having high risk for OSA and divided into the OSA group, otherwise into the non-OSA group.

^†^N represents sample size for non-OSA group or for OSA group.

^‡^Adjustment for age, sex, education, marital status, diabetes, and dyslipidemia.

^ξ^Additional adjustment for body mass index.

e-Table 6 Association between healthy lifestyle score and OSA risk by using median in place of WHO recommended cut-off value of LTPA in generating HLS

| Healthy lifestyle score | N^*^ | |  | Effect | | |
| --- | --- | --- | --- | --- | --- | --- |
|  | Non-OSA group | OSA group |  | Unadjusted OR (95% CI) | Adjusted OR (95% CI) ^†^ | Adjusted OR (95% CI) ^‡^ |
| 0-3 | 1109 | 377 |  | 1.00 | 1.00 | 1.00 |
| 4 | 1583 | 385 |  | 0.72 (0.61, 0.84) | 0.81 (0.69, 0.96) | 0.83 (0.69, 0.99) |
| 5 | 2492 | 477 |  | 0.56 (0.48, 0.66) | 0.71 (0.60, 0.84) | 0.80 (0.67, 0.96) |
| 6 | 2179 | 324 |  | 0.44 (0.37, 0.52) | 0.56 (0.47, 0.67) | 0.74 (0.61, 0.90) |
| 7 | 744 | 63 |  | 0.25 (0.19, 0.33) | 0.33 (0.24, 0.44) | 0.61 (0.45, 0.83) |
| *P* for trend |  |  |  | <0.001 | <0.001 | <0.001 |
| Every 1-point increment |  |  |  | 0.77 (0.74, 0.80) | 0.82 (0.78, 0.86) | 0.90 (0.86, 0.95) |

^*^ N represents sample size for non-OSA group or for OSA group.

^†^ Adjustment for age, sex, education, marital status, diabetes, and dyslipidemia.

^‡^ Additional adjustment for body mass index.

e-Table 7 Sensitivity analysis on the association between healthy lifestyle score and OSA risk after excluding participants aged 65 years and above

| Healthy lifestyle score | N^*^ | |  | Effect | | |
| --- | --- | --- | --- | --- | --- | --- |
|  | Non-OSA group | OSA group |  | Unadjusted OR (95% CI) | Adjusted OR (95% CI) ^†^ | Adjusted OR (95% CI) ^‡^ |
| 0-3 | 587 | 233 |  | 1.00 | 1.00 | 1.00 |
| 4 | 929 | 237 |  | 0.64 (0.52, 0.79) | 0.73 (0.59, 0.91) | 0.68 (0.54, 0.86) |
| 5 | 1756 | 357 |  | 0.51 (0.42, 0.62) | 0.66 (0.54, 0.81) | 0.71 (0.57, 0.89) |
| 6 | 2145 | 284 |  | 0.33 (0.27, 0.41) | 0.47 (0.38, 0.58) | 0.59 (0.47, 0.75) |
| 7 | 947 | 57 |  | 0.15 (0.11, 0.20) | 0.23 (0.17, 0.32) | 0.41 (0.29, 0.57) |
| *P* for trend |  |  |  | <0.001 | <0.001 | <0.001 |
| Every 1-point increment |  |  |  | 0.70 (0.67, 0.73) | 0.77 (0.73, 0.81) | 0.85 (0.80, 0.90) |

^*^ N represents sample size for non-OSA group or for OSA group.

^†^ Adjustment for age, sex, education, marital status, diabetes and dyslipidemia.

^‡^ Additional adjustment for body mass index.

e-Table 8 Sensitivity analysis on the association between healthy lifestyle score and OSA risk after excluding participants with BMI less than 18.5 kg/m^2^

| HLS | N^*^ | |  | Effect | | |
| --- | --- | --- | --- | --- | --- | --- |
|  | Non-OSA group | OSA group |  | Unadjusted OR (95% CI) | Adjusted OR (95% CI) ^†^ | Adjusted OR (95% CI) ^‡^ |
| 0-3 | 655 | 272 |  | 1.00 | 1.00 | 1.00 |
| 4 | 1142 | 301 |  | 0.63 (0.52, 0.77) | 0.70 (0.57, 0.85) | 0.68 (0.55, 0.83) |
| 5 | 2242 | 512 |  | 0.55 (0.46, 0.65) | 0.67 (0.55, 0.80) | 0.71 (0.58, 0.86) |
| 6 | 2641 | 433 |  | 0.39 (0.33, 0.47) | 0.51 (0.42, 0.62) | 0.62 (0.50, 0.76) |
| 7 | 1069 | 95 |  | 0.21 (0.17, 0.27) | 0.29 (0.22, 0.38) | 0.48 (0.36, 0.63) |
| *P* for trend |  |  |  | <0.001 | <0.001 | <0.001 |
| Every 1-point increment |  |  |  | 0.74 (0.71, 0.77) | 0.79 (0.76, 0.83) | 0.87 (0.83, 0.92) |

^*^ N represents sample size for non-OSA group or for OSA group.

^†^ Adjustment for age, sex, education, marital status, diabetes and dyslipidemia.

^‡^ Additional adjustment for body mass index.
